# Psoriatic Arthritis Is an Indicator of Significant Renal Damage in Patients with Psoriasis: An Observational and Epidemiological Study

**DOI:** 10.1155/2017/5217687

**Published:** 2017-03-22

**Authors:** Abidullah Khan, Iqbal Haider, Maimoona Ayub, Mohammad Humayun

**Affiliations:** KTH, Peshawar, Pakistan

## Abstract

*Background*. Psoriasis affects joints in around 30% of the patients. Recent studies have demonstrated an increased risk of essential hypertension, ischemic heart disease, and stroke in psoriatic patients. However, the prevalence of renal disease in patients with psoriasis has not been evaluated properly.* Objectives*. Objectives were to evaluate renal functions in patients with psoriasis and to assess any possible relationship of renal failure with psoriasis and psoriatic arthritis.* Methods*. In this cross-sectional study, 30 participants were recruited into the following three groups: group-A, psoriatic arthritis; group-B, psoriasis without arthritis; and group-C, healthy subjects. Renal function tests were performed for every participant of each group. The data was analyzed by using SPSS version 16. Chi-squared and one-way ANOVA tests were applied, considering a *P* value of less than 0.05 as a standard criterion.* Results*. Serum creatinine, urea, and phosphate were the highest in group-A, higher in group-B, and normal in group-C, *P* < 0.05. Similarly, GFR was the lowest in group-A, lower in group-B, and normal in group-C. The difference in mean GFR values was statistically significant, *F*(2) = 355, *P* < 0.001. Moreover, proteinuria (gm/day) was seen in 96.7% of the patients with psoriatic arthritis, (*M* = 1.18 ± 0.55, *P* < 0.05) against 10% of the psoriatic patients without arthritis (*M* = 0.41 ± 0.10, *P* < 0.05).* Conclusion*. Derangement of renal function is more prevalent in psoriatic patients, especially in those with concomitant psoriatic arthritis. Therefore, each psoriatic patient must be routinely screened for an underlying renal failure.

## 1. Introduction

Psoriasis which affects around 5% of the general population is a chronic autoimmune inflammatory condition of the skin and/or joints. This disease peaks in adulthood and is one of the common causes of increased morbidity worldwide [1, 2]. Recent studies have demonstrated a link between psoriasis and other systemic disorders like diabetes mellitus, metabolic syndrome, cardiovascular, and cerebrovascular diseases [1, 3–6]. The pathogenesis of association of psoriasis with these disorders is attributed to a chronic inflammatory state, driven by activated T-cells and cytokines such as, tumor necrosis factor-*α* (TNF-*α*) [1]. Although, a link between renal impairment and psoriasis has been proposed on the basis of case reports of kidney disease in patients with psoriasis, the association between the two entities still remains largely unclear [7].

Studies examining the possible association between psoriasis and kidney disease have shown paradoxical results. In a study by Yang et al., derangement of renal function was more prevalent in patients with severe psoriasis than in the age and sex matched controls [8]. Similarly, other studies demonstrated a greater incidence of proteinuria and elevated creatinine in patients suffering from psoriasis [9–12]. However, contrarily to the aforementioned research work, other studies failed to show a link between psoriasis and the renal involvement [13–15].

Although, the risk of renal failure in patients with psoriasis has been studied occasionally, renal involvement in subjects with psoriatic arthritis has scarcely been evaluated. Therefore, we investigated the risk of renal involvement in patients with psoriasis, generally, and in those with arthritis psoriatica, specifically. We hypothesized that renal failure would be more frequent in patients with psoriatic arthritis than in those without arthritis. Similarly, we hypothesized that deranged renal function would be more prevalent in patients with psoriasis without arthritis than the age and sex matched normal population.

## 2. Methods

This observational study was conducted in the departments of Dermatology of the three tertiary level hospitals of Peshawar, Pakistan, between February and December 2016, and included 90 subjects. Five of these participants, diagnosed with psoriatic arthritis, were recruited from Medical Unit “A” of Khyber Teaching Hospital (KTH), Peshawar, Pakistan, as well. The participants were divided into three equal groups as follows: group-A, patients with psoriasis and concomitant psoriatic arthritis; group-B, patients with psoriasis without arthritis; and group-C, healthy individuals without either psoriasis or psoriatic arthritis. This study was approved by the relevant hospital's ethics' review board. Furthermore, an informed written consent was obtained from every participant before the conduction of this study.

The inclusion criteria included (1) any gender, (2) age range of 18–70 years, (3) patients with psoriasis and/or psoriatic arthritis irrespective of severity of psoriasis itself for inclusion in either of group-A or group-B, and (4) thirty, age and sex matched healthy individuals for comparison in group-C, as a control group. The diagnosis of psoriasis was made on the basis of clinical grounds, expert opinion of the senior dermatologist, and/or skin biopsy in difficult cases. Similarly, patients were labeled as having psoriatic arthritis on the basis of clinical suspicion and detailed assessment by a senior rheumatologist and/or physician, supported by imaging and synovial fluid analysis in difficult cases.

Those who had preexisting kidney disease were excluded from the study. Similarly, those with risk factors for renal disease like hypertension, hyperlipidemia, diabetes, smoking, lupus, renovascular stenosis, recurrent urinary tract infections (UTIs), vasculitides like Wegner's granulomatosis or Churg-Strauss syndrome, and polyarteritis nodosa were also excluded from the study. Those with a history of intake of nonsteroidal anti-inflammatory drugs (NSAIDs), antibiotics like aminoglycosides, and other nephrotoxic medications in the preceding three months were also excluded. Moreover, patients with psoriasis who either previously received or were currently on potentially nephrotoxic medications for their disease like disease modifying antirheumatic drugs (DMARDs), biologic treatment, and so forth were also excluded from the study.

Initially, 200 individuals were assessed for their suitability in the final study. However, only 90 of them fulfilled the inclusion/exclusion criteria and were, thus, the subjects of final study. Each included participant was worked up with a detailed history, clinical examination, and a thorough review of the previous medical record. Serum creatinine, urea, and urinary protein estimation were done through enzymatic colorimetric assays on the automated biochemistry analyzer, ABX Pentra-400, Horiba Medical, France. Moreover, serum calcium and phosphate levels were measured through direct potentiometry through ion-selective electrodes (ISE) method by using the same biochemistry analyzer. Blood hemoglobin (Hb) was determined by using spectrophotometry on an automated 19-parameter hematology analyzer, Counter-19, provided by Weiner lab, Argentina. Similarly, glomerular filtration rate (GFR) and 24-hour urinary protein were measured for every participant. GFR was calculated by using Modification of Diet in Renal Disease (MDRD) criteria. Those who had significant proteinuria, defined as more than 0.5 gm protein per day, were subjected to a renal biopsy for histological evaluation.

All the data was obtained on a structured questionnaire specifically designed for this purpose. The questionnaire included questions regarding demography, disease duration, past medical and surgical history, drug history, and the study variables mentioned above. All the data was entered into and analyzed by the SPSS version 16. One-way ANOVA, with Turkey-HSD, and Chi-squared tests were used for the statistical analysis. A* P* value of less than 0.05 was considered as a standard criterion.

## 3. Results

Mean age of all the 90 participants was 35.71 ± 6.90 years. The male population made 66.70% of the sample while females were 33.30%. Majority of individuals with psoriasis or psoriatic arthritis worked in occupations with maximum sun exposure (more than 8 hours/day), such as outdoor hard labor, public driving, and farming. However, most of the healthy participants had minimum sun exposure. Occupation, age, and genderwise descriptive statistics for each group are given below (Table 1).

The results of Chi-squared test revealed that although psoriasis was more common in people belonging to occupations with maximum sun exposure (more than 8 hours/day), such as laborers, drivers, and farmers (*N* = 19 (37.3%), *Z*-score = 0.9), than those who were least exposed to sun, such as bankers and health professionals (*N* = 11 (28.2%), *Z*-score = 0.9), the difference was not significant statistically,* P* > 0.05. Nevertheless, psoriatic arthritis was more prevalent in patients with maximum sun exposure than those less exposed to sun, and at a statistically significant level, Chi-squared (2) = 18.19,* P* < 0.001. The likelihood ratio (LR) for having psoriatic arthritis in patients with maximum sun exposure was statistically significant as well, LR (2) = 18.91,* P* < 0.001. Similarly, although, both psoriasis and psoriatic arthritis were seen to be more prevalent in males, but the impact of gender on having psoriasis or psoriatic arthritis was not found to be significant statistically, Chi-squared (2) = 0.30,* P* = 0.86, and LR (2) = 0.30,* P* = 0.86, respectively.

**Figure 1:**
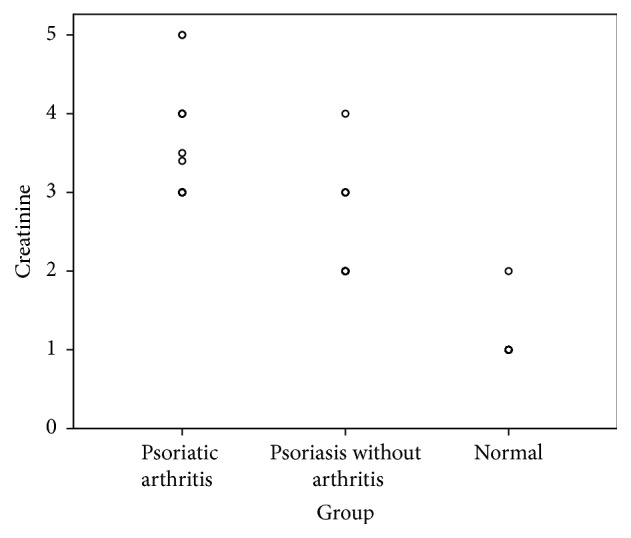
Scatter dot plot demonstrating groupwise distribution of serum creatinine (mg/dl).

**Figure 2:**
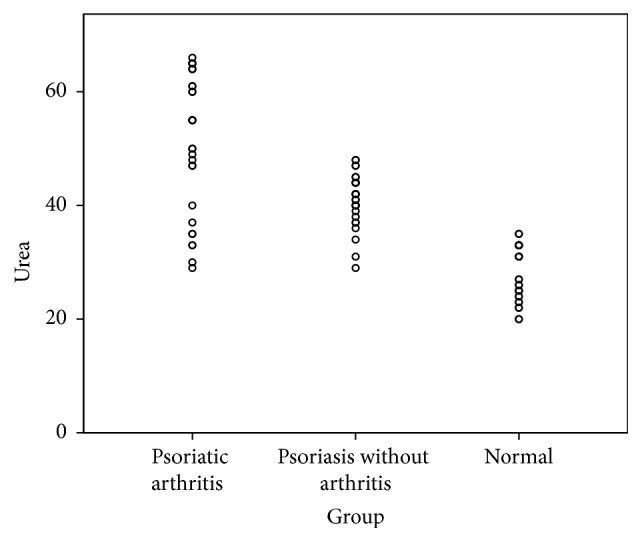
Scatter dot plot demonstrating groupwise distribution of serum urea (mg/dl).

Assessment of renal function revealed that patients with psoriatic arthritis had significantly elevated creatinine [(mg/dl), (*M* = 3.36 ± 0.60,* P* < 0.001)] and urea [(mg/dl), (*M* = 51.43 ± 12.29,* P* < 0.001)]. Similarly, patient with psoriasis but without concomitant psoriatic arthritis still had significant derangement of creatinine (*M* = 2.30 ± 0.53, *P* < 0.001) and urea (*M* = 41.03 ± 4.62, *P* < 0.001), compared with the normal control group (Table 2). Groupwise distribution of serum creatinine and urea is given below (Figures 1 and 2).

**Figure 3:**
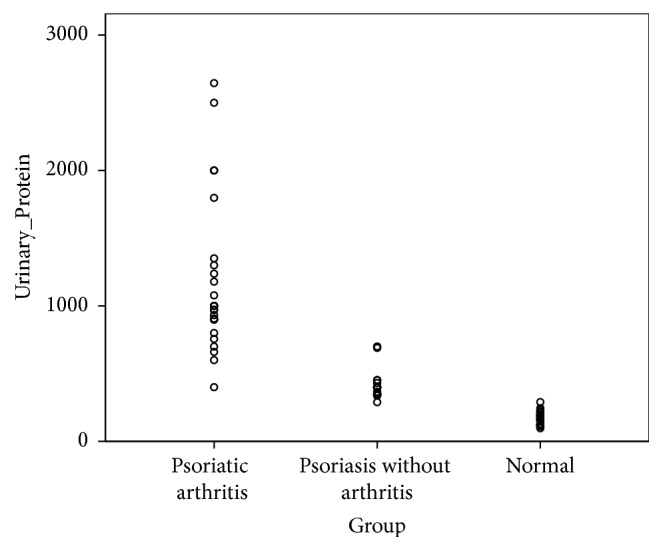
Scatter dot plot demonstrating groupwise distribution of daily proteinuria (mg/day).

Nevertheless, clinically significant proteinuria, defined as more than 500 mg of protein in 24-hour urine sample, was seen in 96.70% (*n* = 29) of the participants of group-A (patients with both psoriasis and psoriatic arthritis), 10% (*n* = 3) of the participants of group-B, (patients with psoriasis without arthritis), and none of the participants of group-C (the healthy subjects), as demonstrated below (Figure 3). All the 32 patients (35.6%), with clinically significant proteinuria, had renal biopsies; the results are as shown (Table 3). The predominant histological pattern of renal damage was that of membranous glomerulonephritis (MGN). No one had any evidence of renal tubular damage.

**Figure 4:**
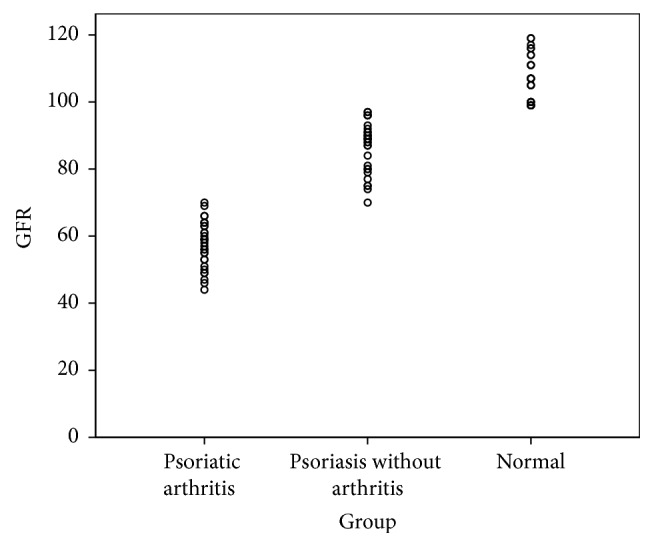
Scatter dot plot demonstrating groupwise distribution of e-GFR (ml/min).

**Figure 5:**
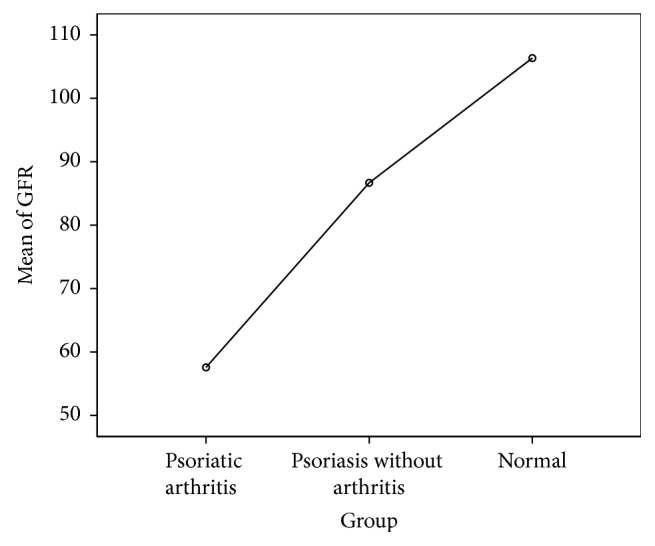
Mean plot demonstrating variation in e-GFR (ml/min) across the three study groups.

As can be seen in Table 2, the renal functions were impaired more in patients with psoriatic arthritis than those with psoriasis only. Similarly, the healthy controls had normal renal parameters. Furthermore, the GFR (ml/minute) was the lowest in group-A (*M* = 57.57, SD = 6.90), intermediate in group-B (*M* = 86.67, SD = 7.49), and the highest in group-C (*M* = 106.33, SD = 6.95). In order to compare mean values of GFR across these three groups, one-way ANOVA was run. The assumption of homogeneity of variance was tested and was found tenable; Levene's statistic (2, 87) = 0.10,* P* = 0.90. Similarly, the assumption of normality was satisfied (skewness = −0.19, kurtosis = −1.21). The results of one-way ANOVA demonstrated a statistically significant difference in mean GFR of the three study groups, *F*(2) = 355,* P* < 0.001. Post hoc analysis via Turkey-HSD showed that all the three groups were different from each other at a statistically significant level,* P* < 0.001 (Figures 4 and 5). Thus it can be concluded that there is enough evidence of significant renal impairment in patients with either psoriatic arthritis or psoriasis, compared with the age and sex matched unaffected individuals. This manifests in the form of raised creatinine, urea, and phosphate and a concomitant decline in GFR, Hb, and calcium,* P* < 0.05 (Table 2).

## 4. Discussion

We observed that both psoriasis and psoriatic arthritis are common in men in their 3rd to 4th decade of life. Moreover, they are more prevalent in persons exposed maximally to sunlight, such as farmers, laborers, sewerage worker, and drivers, and are relatively uncommon in people who remain or work indoors like bankers and health professionals. Our study demonstrated that patients with psoriatic arthritis are more likely to have deranged renal functions than those with psoriasis but without concomitant joint disease. However, such patients still have more incidence of renal disease than the normal people. Although, clinically significant proteinuria is more common in arthritis psoriatica, its prevalence in patients with psoriasis only is still significantly higher than the healthy controls. The most common renal abnormality in such people is that of membranous glomerulonephritis (MGN).

It is worth mentioning that a limited number of studies have demonstrated a link between psoriasis and kidney disease. The most commonly encountered renal problems in patients with psoriasis are IgA nephropathy, focal segmental glomerulosclerosis (FSGS), and membranous nephropathy [9, 16–19]. It is noteworthy that membranous glomerulonephritis (MGN) and focal segmental glomerulosclerosis (FSGS) were the most common histological patterns on renal biopsies in our study group.

There is some emerging evidence that immunologic mechanisms such as chronic T-cell activation and increased levels of immune complexes and cytokines cause glomerular injury in psoriasis [7, 20]. However, other studies have demonstrated that direct tubular injury resulting from hyperuricemia in people with psoriasis could be another probable mechanism [21]. It should be noted that although we observed high uric acid levels in psoriatic patients with concomitant renal involvement, none had histologically proven renal tubular necrosis.

The extent of psoriasis is an independent risk factor for systemic involvement. In a study by Kurd and Gelfand, chronic kidney disease was more prevalent in patients with moderate-severe psoriasis than in those with mild disease [2]. However, it must be noted that one of the limitations of our study was the nonaccounting for the extent of psoriasis in terms of body surface area (BSA) involved.

Gelfand et al. observed that the risk of kidney disease was higher in the young than the elderly psoriatic patients [3]. This finding is in sharp contrast to our observation of a positive association between the risk of renal involvement and age of the patients. However, our finding of positive association between increasing age and the risk of renal disease in patients with psoriasis is supported by other relevant studies [22]. It has been argued that aging leads to a higher activity of inflammation-associated transcription factors, namely, NF*κ*B, STAT1, and STAT3. This ultimately leads to an escalated action of TNF- *α*, causing renal injury [23]. Moreover, one recent study concluded that psoriasis accounted for one extra case of chronic kidney disease per 134 patients per year in patients aged 40–50, and in those aged 50–60, the incidence was even higher at one additional case per 62 patients per year [24].

Although we excluded all those patients who had received potentially nephrotoxic drugs for the treatment of psoriasis, Corden et al. reported a case of acute tubular necrosis (ATN), developing secondary to the topical use of vitamin-D in a psoriatic patient [25]. Hence, studies recommend rigorous monitoring of the renal function in patients treated for psoriasis with any potentially nephrotoxic medication [25, 26].

It is worth mentioning that the results of this study were according to our expectations. This study demonstrated an increased incidence of renal impairment in psoriasis and especially in psoriatic arthritis. However, this study still had a few limitations. The foremost was that of a smaller sample size. Second limitation was the cross-sectional study design. Thirdly, not all the patient had renal histological examination. Therefore, we recommend future cohort studies to throw more light on the possible link of psoriasis and psoriatic arthritis with kidney disease.

## 5. Conclusion

Renal function is impaired in people with psoriasis and psoriatic arthritis. Furthermore, psoriatic arthritis is an independent predictor of renal damage in patients with psoriasis. Therefore, every patient with psoriasis and/or psoriatic arthritis should be screened for an underlying renal damage.

## Figures and Tables

**Table 1:** Group specific demographic details.

Variable	Group-A	Group-B	Group-C
Males	63.3%	66.7%	70%
Females	36.7%	33.3%	30%
Occupations with maximum sun exposure (>8 hours/day)	80%	63.3%	26.7%
Occupations with minimum sun exposure (<8 hours/day)	20%	36.7%	73.3%
Age (years)	36.43 ± 6.26	34.6 ± 6.68	36.10 ± 7.76

Group-A: patients with psoriatic arthritis, group-B: psoriasis without arthritis, and group-C: healthy controls.

**Table 2:** An overview of the different parameters of renal functions.

Variable	Group-A	Group-B	Group-C	*P* value
Creatinine (mg/dl)	3.36 ± 0.60	2.30 ± 0.53	1.03 ± 0.18	<0.001
Urea (mg/dl)	51.43 ± 12.29	41.03 ± 4.62	28.07 ± 4.77	<0.001
GFR (ml/minute/1.73 m^2^)	57.57 ± 6.90	86.67 ± 7.49	106.33 ± 6.95	<0.001
Urinary protein (mg/day)	1182 ± 549	416 ± 102	170 ± 46	<0.001
Phosphate (mg/dl)	5.50 ± 0.63	3.97 ± 0.49	2.87 ± 0.57	<0.001
Calcium (mg/dl)	8.50 ± 0.63	9.93 ± 0.64	10.37 ± 0.61	<0.001
Hemoglobin (gm/dl)	11.20 ± 1.18	12.47 ± 0.57	14.33 ± 0.99	<0.001
Uric acid (mg/dl)	7.7 ± 0.91	6.9 ± 1.11	5.3 ± 1.23	<0.05

Group-A: patients with psoriatic arthritis, group-B: psoriasis without arthritis, group-C: healthy controls, and GFR: glomerular filtration rate.

**Table 3:** An overview of the pattern of histological involvement of the kidneys in 32 patients with proteinuria of more than 0.5 gm/day.

Histology	Group-A	Group-B	Total (*n*)
Membranous glomerulonephritis (MGN)	25 (92.6%)	2 (7.4%)	27
Focal segmental glomerulosclerosis (FSGS)	3 (75%)	1 (25%)	4
Membranoproliferative glomerulonephritis (MPGN)	1 (100%)	0 (0%)	1
	29	3	32

## Abbreviations

GFR: Glomerular filtration rate
MGN: Membranous glomerulonephritis
FSGS: Focal segmental glomerulosclerosis
Hb: Hemoglobin
ATN: Acute tubal necrosis
DMARDs: Disease modifying antirheumatic drugs
TNF-*α*: Tumor necrosis factor-*α*
MDRD: Modification of diet in renal disease.
